# Ultralong π-phase shift fiber Bragg grating empowered single-longitudinal mode DFB phosphate fiber laser with low-threshold and high-efficiency

**DOI:** 10.1038/s41598-018-31528-w

**Published:** 2018-09-03

**Authors:** Man Jiang, Pu Zhou, Xijia Gu

**Affiliations:** 1 College of Optoelectronic Science and Engineering, National University of Defense Technology, Changsha, 410073 China; 2 Department of Electrical and Computer Engineering, Ryerson University, 350 Victoria St., Toronto, Ontario, M5B 2K3 Canada

## Abstract

Phosphate glass fiber is one of the candidates for building compact fiber lasers because of its capability of high-concentration of rare-earth ions doping in fiber core. Nevertheless, it is challenging for the integration of UV-written intra-core fiber Bragg gratings into the fiber laser cavity due to the low photosensitivity of phosphate glass fiber. The research presented in this paper will focus on demonstration of UV-written Bragg gratings in phosphate glass fiber and its application in direct-written short monolithic single-frequency fiber lasers. A 5-cm-long strong π-phase shift Bragg grating structure is direct-inscribed into the Er/Yb co-doped gain fiber using an excimer laser. The fiber laser device emits output power of 10.44 mW with a slope efficiency of 21.5% and the threshold power is about 42.8 mW. Single-longitudinal mode operation is validated by radio frequency spectrum measurement. Moreover, the output spectrum at the highest power shows an excellent optical signal to noise ratio of about 70 dB. These results, to the best of our knowledge, show the lowest power threshold and highest efficiency among the reports that using the same structure to achieve single-longitudinal mode laser output.

## Introduction

In the past few years, single-frequency lasers have attracted a lot of interest for their important applications and developments in different fields such as coherent telecommunications, LIDAR, high resolution sensing, atom frequency standard, etc.^1–6^ Distributed feedback (DFB) fiber lasers^7–9^ as well as distributed Bragg reflector (DBR) fiber lasers^10–12^ are two of the most common routes to achieve single-frequency lasing. Both DFB and DBR fiber lasers are constructed based on short-length linear cavity, a few cm in length at most, to enlarge longitudinal-mode spacing and hence achieve robust single-frequency operation. The cavity of a DBR single-frequency fiber laser combines a pair of narrowband fiber Bragg gratings (FBGs) with a short piece of gain fiber with high gain coefficient. A DFB fiber laser is composed of a piece of FBG written directly in the active fiber. In general, the FBG has been introduced a phase change in the grating area that results in an ultra-narrow spectral filter to achieve single-mode oscillation. Such a DFB laser exhibit numerous excellent properties including low insertion loss, low-noise characteristics and extremely narrow linewidth output with compact structure.

In the 1.5 μm regime, to implement a stable single-frequency DFB fiber laser operation, silica fibers doped with rare earth ions are routinely used as the gain medium of fiber laser. However, the power that a single-frequency DFB fiber laser can provide is very limited due to the short length of active fiber. Although one could increase the output power by multi-stage amplification^13–15^, the signal to noise radio (SNR) of single-frequency fiber laser will be decreased at the same time^4^. Thus, it is a prerequisite of these sources to have high-concentration of rare-earth ions doping in the core of the active fiber to facilitate high pump absorption coefficient and hence increase the output power as well as maintain the SNR of single-frequency fiber laser. In fact, high doping level of rare earth ions in the phosphate glasses have been successfully used for promoting fiber gain in lasers with a cavity as short as several centimeters^16,17^. Compared with silica fibers, phosphate fibers have higher solubility level of rare-earth ions which can be employed for further doping. Moreover, phosphate fibers have weaker cluster formation which causes concentration quenching. However, due to the lacking sufficient photosensitivity, phosphate fiber is difficult to be directly inscribed an intracore Bragg grating. The main approach used to demonstrate phosphate fiber laser is to employ external resonator mirrors, for example, DBR fiber laser in which Bragg gratings written in silica fibers fusion spliced to phosphate fiber. This design, despite its success, would require special approaches of splicing the two different kinds of fibers to decrease optical losses^18,19^.

In recent years, A. Schülzgen *et al*. successfully presented and demonstrated the direct writing of strong Bragg grating in both Er/Yb co-doped and undoped phosphate glass fibers using UV irradiation from an ArF excimer laser at 193 nm^20,21^. Furthermore, by using the 3.5-cm-long Bragg grating which was direct inscribed into the Er/Yb co-doped phosphate fiber, they achieved single-frequency DFB fiber laser with output power up to 165 mW and 550 mW successively by employing a cladding pumping scheme^22,23^. Moreover, the power thresholds of these two lasers were 1 W and 200 mW, and the slope efficiency were 1.3% and 12%, respectively. In 2014, L. Xiong *et al*. from the same group, using a core pumping scheme demonstrated a DFB fiber laser that achieved output power of 27.5 mW with a lower power threshold of ~ 132 mW, and the slope efficiency was 8.82%^24^.

In this paper, a 5-cm-long strong π-phase shift Bragg grating structure is direct inscribed into the Er/Yb co-doped phosphate fiber using excimer laser, and a DFB fiber laser with 1534 nm single-longitudinal mode output has been achieved. The fiber laser device emits output power of 10.44 mW with a slope efficiency of 21.5% and the threshold power is about 42.8 mW. Moreover, the output spectrum at the highest power shows an excellent optical signal to noise ratio of about 70 dB. These results, to the best of our knowledge, show the lowest power threshold and highest efficiency among the reports that using the same structure to achieve single-longitudinal mode laser output.

## Results

**Figure 1:**
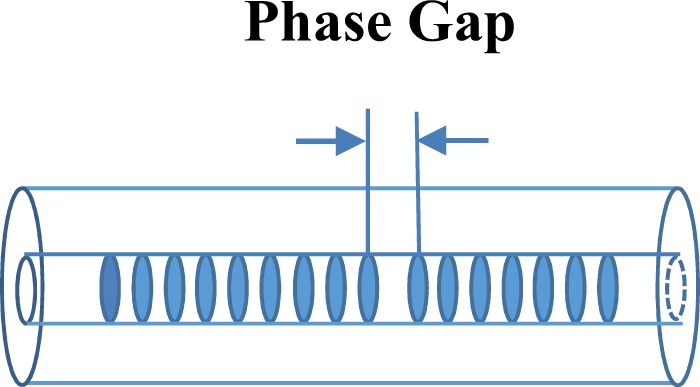
Schematic diagram of π-phase shift grating structure.

A 5-cm-long phase-shift phase mask is used to direct inscribe strong π-phase shift Bragg grating structure into the Er/Yb co-doped phosphate glass fiber. An excimer laser (Lumonics, model: PM844) with the KrF gas mixture, operating at 248 nm is used as a UV source. The typical energy density of the 248 nm pulses at the fiber is approximately 0.09 J/mm^2^ per pulse. The pulse repetition rate is set at 30 Hz and a typical exposure time is 1.5 min to 2 min. The active fiber used is an Er/Yb co-doped fiber with a core diameter of 4.66 µm. Yb absorption at 980 nm is about 2 dB/cm and Er absorption at 1530 nm is about 30 dB/m. A Ge-doped photosensitive ring with a diameter of 16 µm surrounds the fiber core. The π–phase shifted FBG is inscribed in this ring. The Er/Yb co-doped fiber is loaded in a pressurized hydrogen tube of 100 atmosphere pressure for 12 days to increase its photosensitivity. And in order to smooth the reflection spectrum of the grating, the technology of apodization has been used into the process of FBG inscription. The FBG is annealed at 150 °C for 15 hours after inscription to ensure its long-term stability. A DFB laser cavity has been achieved thereon. The phase mask is a uniform mask with the grating pitch of 1059 nm. Moreover, a 50 μm gap is in the middle of the phase mask, which splits the mask into two uniform sections. The grating structure of the phase mask is replicated into the active phosphate fiber during UV irradiation, and Fig. 1 illustrates the schematic of the FBG. Using a π-phase shifted phase mask offers a better way of locating the position of the phase gap along the FBG since the 50 µm phase gap would show as a vertical dark line when the laser beam was projected on a screen after passing through the phase mask. With that the phase gap can be positioned either in the center or off center to increase its output from one end. In addition, the π-phase shifted phase mask gives an improved apodization spectral profile in comparison with the other methods of generating a π-phase in the FBGs such as the post-processing method.

**Figure 2:**
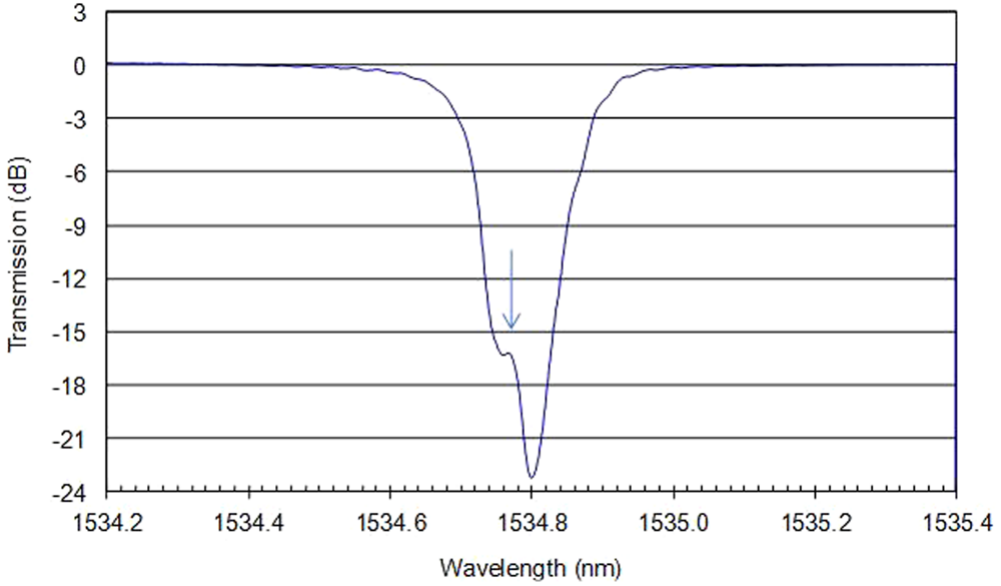
The measured transmission spectrum of the DFB grating structure.

By the optical spectrum analyzer (ANDO Corp. model AQ6317), the experimental transmission spectrum of the π-phase shift Bragg grating in the doped phosphate fiber is shown in Fig. 2. In the measured spectrum, the transmission dip of the DFB grating structure reached −23.14 dB at the peak wavelength of 1534.771 nm. Moreover, the −3 dB transmission bandwidth of the grating is only 0.185 nm result from the 5-cm-long phase mask. Due to the limited optical spectrum analyzer resolution of 0.01 nm, no clear resonant Fabry-Perot transmission peak is shown inside the grating bandwidth, and the practical position of the FP peak is arrowed on the figure. Reasonable agreement is found between the measured and simulated transmission spectra of this DFB grating structure. And the 50 μm gap in the grating lead only one FP resonance exists within the grating bandwidth as shown in the spectral figure. This result ensures that the DFB laser can operate at robust single longitudinal mode without mode hopping.

**Figure 3:**
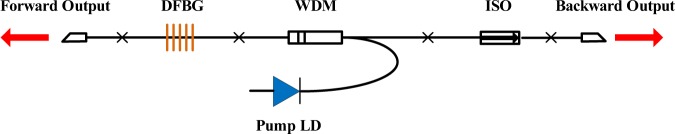
Schematic diagram of the DFB laser experiment setup.

Then we use the π-phase shift Bragg grating aforementioned to demonstrate the DFB phosphate fiber laser and Fig. 3 illustrate the schematic of this DFB laser. A 6-cm-long Er/Yb co-doped phosphate fiber with DFBG in the core is used as the gain medium and has been spliced to the standard telecommunication fiber pigtails. The DFB laser is core-pump by a 980 nm laser diode (LD) with maximum output power of 105 mW through a WDM coupler. The 5-cm-long DFB grating structure has decreased the length of laser oscillator and increased the space of output longitudinal mode effectively. Moreover, the rest part of the doped fiber will absorb the remaining pump light and amplify the laser signal. An isolator (ISO) is used in order to prevent the backward signal. In the experiment, define forward direction is the direction same with pump light, and the counter direction is backward.

**Figure 4:**
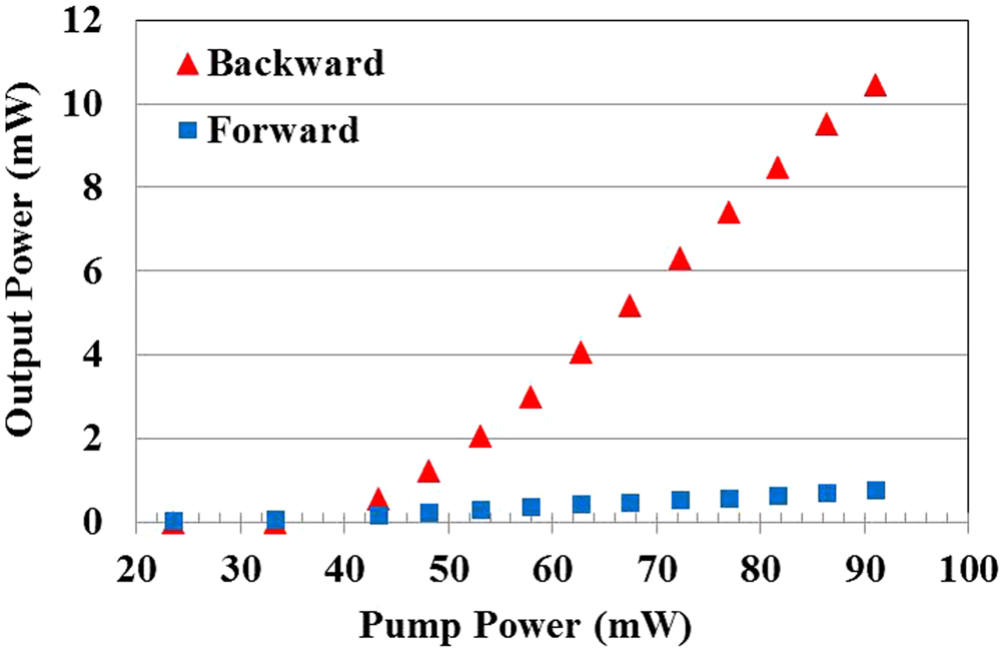
DFB laser output power vs. pump power.

The laser output power from both forward and backward plotted against the pump power for the DFB fiber laser is shown in Fig. 4. The maximum backward output power obtained is 10.44 mW for an incident pump power of 91.1 mW. The threshold power is observed to be 42.8 mW, which is about one third the value of previously reported results^24^. And a slope efficiency of 21.5% has been achieved. This result, to the best of our knowledge, is the highest efficiency among the reports that using the same structure to achieve single-longitudinal mode DFB laser. Moreover, Fig. 4 shows that the backward output power is about two orders higher than the forward output power that means this DFB fiber laser has achieved truly unidirectional output.

**Figure 5:**
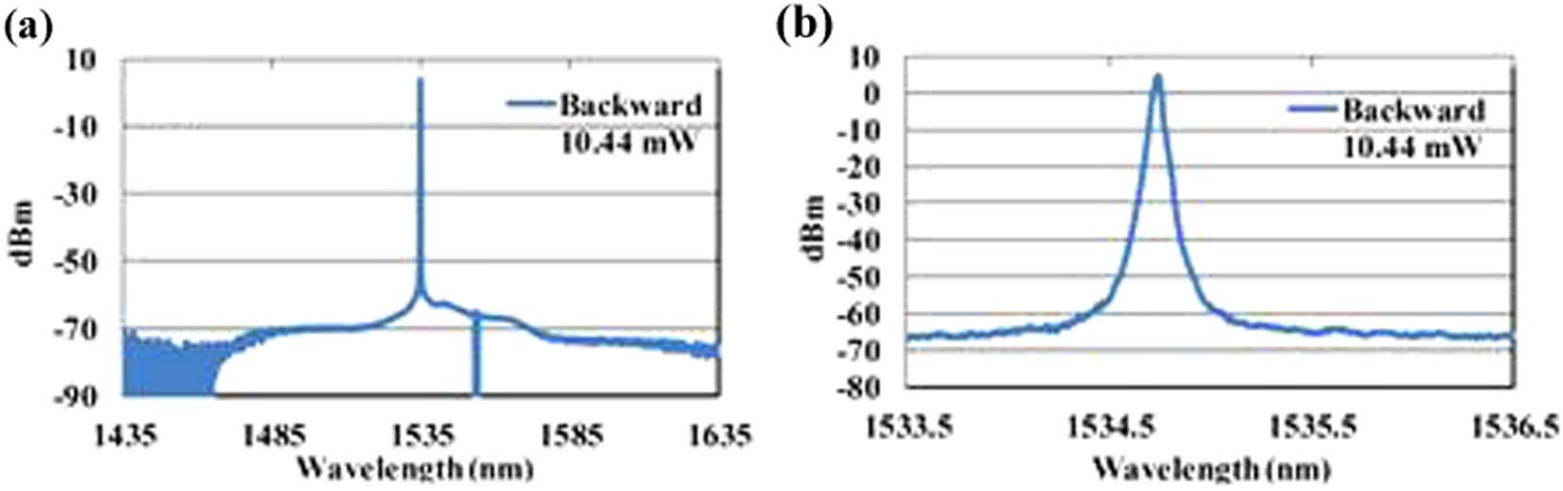
The output spectra from backward of the DFB laser: (**a**) output spectrum within 200 nm bandwidth; (**b**) output spectrum within 3 nm bandwidth.

We also measure the optical spectra of the backward output of the DFB fiber laser operated at output power of 10.44 mW, as shown in Fig. 5. Therein, (a) is the output spectrum measured within 200 nm bandwidth ranges with the optical spectrum analyzer resolution of 0.1 nm, and (b) is the output spectrum measured within 3 nm bandwidth range with the optical spectrum analyzer resolution of 0.01 nm. The SNR of this DFB fiber laser is about 70 dB, and the lasing wavelength is stabilized at 1534.7 nm.

**Figure 6:**
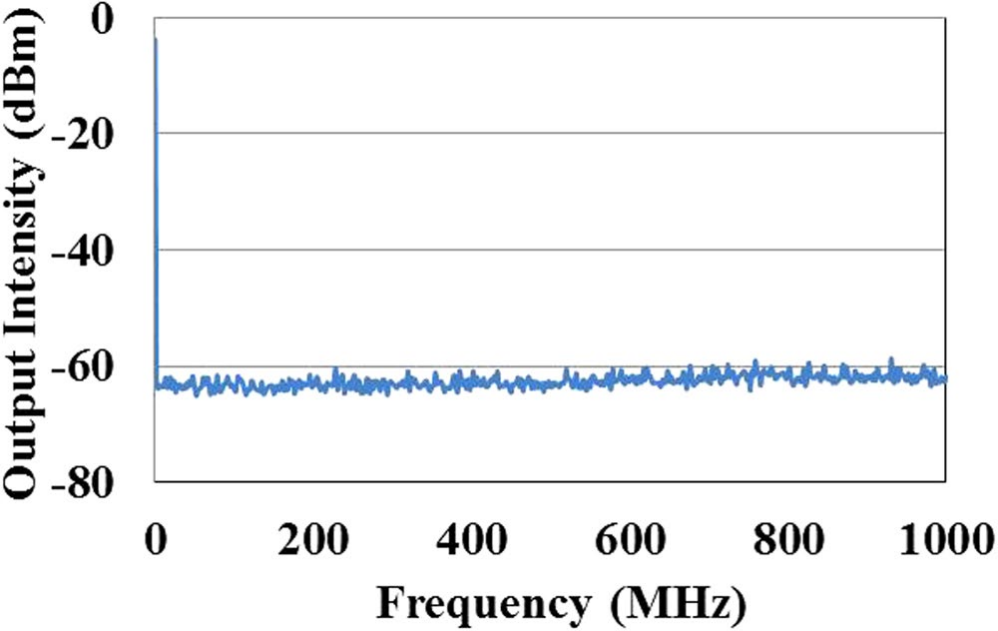
Measured RF result of DFB laser with maximum output power.

The measured radio frequency spectrum (RF) of the DFB fiber laser by the emission spectrometer within 1 GHz range and resolution of 100 kHz is shown in Fig. 6. This result illustrate that the DFB fiber laser has achieved single frequency laser output.

In conclusion, strong π-phase shift Bragg grating structure is direct-inscribed into the Er/Yb co-doped phosphate fiber using excimer laser to build a DFB laser cavity. The DFB fiber laser cavity is achieved by a 5-cm-long strong π-phase shift Bragg grating structure direct inscribed into the Er/Yb co-doped phosphate fiber, and 1534 nm single-longitudinal mode output has been obtained. The fiber laser device emits output power of 10.44 mW with a slope efficiency of 21.5% and the threshold power is about 42.8 mW. Moreover, the output spectrum at the highest power shows an excellent optical signal to noise ratio of about 70 dB. It is worth mentioning that the low power threshold shows the superiority of our DFB grating structure, and the principle of the DFB fiber laser’s design is general and can be applied to other type of fiber lasers or wavelengths. For example, by reasonably design the parameters of DFB grating, it can be used to achieve 1-μm DFB single-frequency fiber laser.

## Discussion

We compared our results and efficiency to other fiber sources in this wavelength band using the same structure with Er/Yb co-doped phosphate fiber. As stated previously, the threshold power and slope efficiency of this DFB laser are 42.8mWand 21.5%, respectively. For cladding pumping scheme^23^, the lowest power threshold is 200 mW and the slope efficiency is 12% by a 3.5-cm-long Bragg grating which build the DFB laser cavity. For core pumping scheme^24^, the lowest power threshold is ~132 mW and the slope efficiency is 8.82%. To demonstrate lower threshold and higher efficiency single-longitudinal mode laser output by DFB laser cavity scheme, longer strong pi-phase shifted FBG is the prerequisite and important element in this paper. Compare to the 3.5-cm-long π-phase shift grating, the 5-cm-long grating would decrease the threshold power and enhance the slope efficiency for a DFB laser cavity. Thus, the research presented in this paper is also focused on the demonstration of UV-written long Bragg gratings in phosphate glass fiber.

According to the coupled mode theory, the reflection properties of a FBG can be regarded as light coupling between counter propagating optical fields guided inside the grating. Thus, the reflectivity of a uniform FBG is calculated from the coupling equation^25,26^ as:

1 $${\rm{R}}({\rm{1}},{\rm{\lambda }})=\frac{{{\rm{\kappa }}}^{2}{\sinh }^{2}\,({\rm{s}}1)}{{{\rm{\Delta }}{\rm{\kappa }}}^{2}{\sinh }^{2}\,({\rm{s}}1)+{{\rm{s}}}^{2}{\cosh }^{2}\,({\rm{s}}1)}$$

Where R is the power reflectivity of the grating, *l* is length of the grating, *λ* is the reflected wavelength, *κ* is the coupling coefficient, ${\rm{\Delta }}\kappa =\kappa -\frac{\pi }{{\rm{\Lambda }}}$ is the detuning wave vector which is the difference between propagation constant at reflected wavelength and that at Bragg wavelength,$\,{\rm{\Lambda }}$ is the nominal period, and *s*^2^ = *κ*^2^−Δ*κ*^2^. Note that the propagation constant *κ* = 2π*n*_*eff*_/*λ*, where the *n*_*eff*_ is the effective index which can be determined from the average fiber core refractive index. The coupling coefficient *κ* is given by

2 $$K=\frac{\pi {\rm{\Delta }}{{n}}_{{mod}}}{\lambda }{\rm{\Gamma }}$$

Where Δ*n*_*mod*_ is the refractive index modulation amplitude, and the confinement factor ${\rm{\Gamma }}$ present the fraction of the fiber mode power contained by the fiber core. In general, for the fundamental mode of a standard single mode fiber in the wavelength region around 1550 nm, ${\rm{\Gamma }}\approx 0.75$.

According to the reflectivity formula given by Eqs (1) and (2), two FBGs have been formation with the same refractive index modulation by employing the phase-shift phase mask aforementioned. Moreover, longer FBG has a higher reflectivity than the shorter FBG under the condition of both having the same refractive index modulation. Therefore, by changing the position of the gap in the FBG would control the reflectivity of these two sections, and hence ensures the output power is predominantly emitted from one end of the DFB laser.

**Figure 7:**
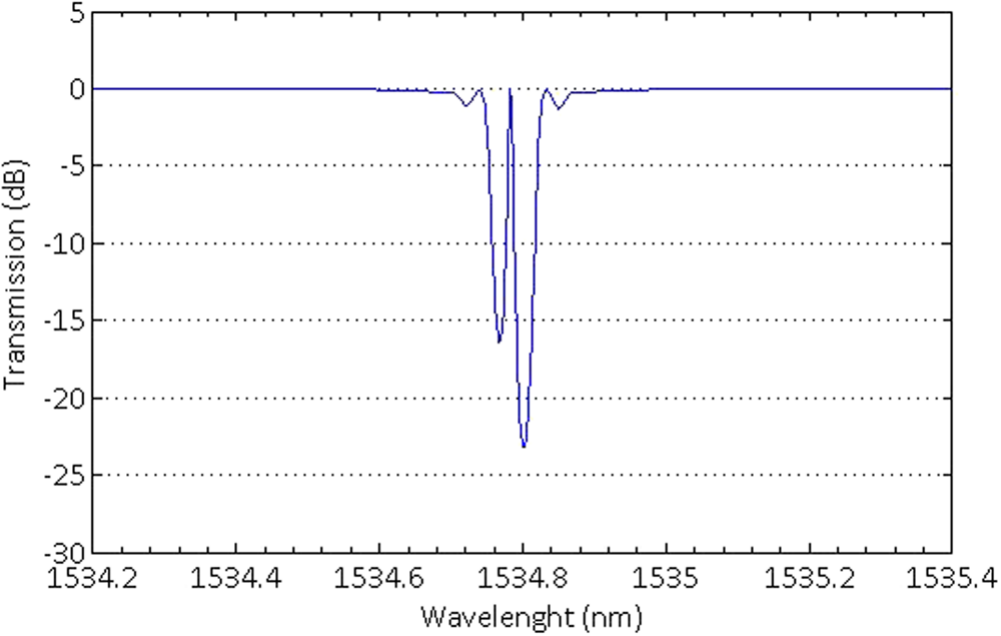
The simulated transmission spectrum of the 5-cm-long π-phase shift grating.

For applications that place high demands on the spectral shape of the grating with high reflectivity, the technology of apodization and complementary which suppress the side lobes in the reflection spectrum by gradually increasing the coupling coefficient with penetration into as well as gradually decreasing on exiting from, has been widely used. In this paper, we employ the super-Gaussian function to realize apodization and complementary for the grating. And for a non-uniform grating, the couple mode equations always being solved by piecewise-uniform approach^27^, in which the grating is divided into a number of uniform pieces. The closed form solution for each uniform piece is combined by multiplying matrixes associated with the pieces. Consequently, for the π-phase shift grating, the results of numerical simulation are shown in Fig. 7. Reasonable agreement is found between the measured and simulated transmission spectra of this DFB grating structure.

## Method

### Measurement Method

An excimer laser (Lumonics, model: PM844) with the KrF gas mixture, operating at 248 nm is used as a UV source. The coating of the fiber, about 55 mm long, is chemically removed before UV exposure. The typical energy density of the 248 nm pulses at the fiber is approximately 0.09 J/mm^2^ per pulse. The pulse repetition rate is set at 30 Hz and a typical exposure time is 1.5 min to 2 min. The fiber is loaded in a pressurized hydrogen tube of 100 atmosphere pressure for 12 days to increase its photosensitivity. All FBGs are annealed at 150 °C for 15 hours after inscription to ensure their long-term stability. An optical spectrum analyzer (ANDO Corp. model AQ6317) with a resolution of 0.01 nm is employed to measure the spectrum of both π-phase shift grating and DFB laser. In order to reduce the measurement error, one power meter is used to measure the output power of both forward and backward output laser. When measuring the spectra of π-phase shift grating, a narrow-linewidth tunable laser in 1.5 μm band is used. We fix the fiber pigtail to one end of fiber grating, and the other end of grating is coupled into OSA. When measuring the spectra of DFB fiber laser, a piece of single mode fiber is used to couple output laser beam into OSA. During the experiments, the signal-to-noise ratios (SNRs) of the DFB laser’s spectra are guaranteed to be higher than ~70 dB. Radio frequency spectrum characteristics of DFB laser is detected by employing an emission spectrometer within 1 GHz range and resolution of 100 kHz.
